# Synergistic cytotoxicity of the CDK4 inhibitor Fascaplysin in combination with EGFR inhibitor Afatinib against Non-small Cell Lung Cancer

**DOI:** 10.1007/s10637-021-01181-8

**Published:** 2021-10-01

**Authors:** Adelina Plangger, Barbara Rath, Maximilian Hochmair, Martin Funovics, Christoph Neumayer, Robert Zeillinger, Gerhard Hamilton

**Affiliations:** 1 Institute of Pharmacology, Medical University of Vienna, Vienna, Austria; 2 Department of Respiratory & Critical Care Medicine, Karl Landsteiner Institute of Lung Research & Pulmonary Oncology, Vienna, Austria; 3 Division of Cardiovascular and Interventional Radiology, Department of Biomedical Imaging and Image-Guided Therapy Medical, University of Vienna, Vienna, Austria; 4 Department of Vascular Surgery, Medical University of Vienna, Vienna, Austria; 5 Molecular Oncology Group, Department of Obstetrics and Gynecology, Medical University of Vienna, Vienna, Austria

**Keywords:** Non-small cell lung cancer, Pleural effusion, Fascaplysin, Afatinib, Cytotoxicity, Protein phosphorylation

## Abstract

In the absence of suitable molecular markers, non-small cell lung cancer (NSCLC) patients have to be treated with chemotherapy with poor results at advanced stages. Therefore, the activity of the anticancer marine drug fascaplysin was tested against primary NSCLC cell lines established from pleural effusions. Cytotoxicity of the drug or combinations were determined using MTT assays and changes in intracellular phosphorylation by Western blot arrays. Fascaplysin revealed high cytotoxicity against NSCLC cells and exhibit an activity pattern different of the standard drug cisplatin. Furthermore, fascaplysin synergizes with the EGFR tyrosine kinase inhibitor (TKI) afatinib to yield a twofold increased antitumor effect. Interaction with the Chk1/2 inhibitor AZD7762 confirm the differential effects of fascplysin and cisplatin. Protein phosphorylation assays showed hypophosphorylation of Akt1/2/3 and ERK1/2 as well as hyperphosphorylation of stress response mediators of H1299 NSCLC cells. In conclusion, fascaplysin shows high cytotoxicity against pleural primary NSCLC lines that could be further boosted when combined with the EGFR TKI afatinib.

## Introduction

Approximately 80% of all lung cancers are of the Non-small Cell Lung Cancer (NSCLC) type that is often detected at an advanced stage and portends a dismal prognosis [1]. The standard first-line therapy employing platinum-based chemotherapy resulted in minor improvements in survival but at the cost of side effects and poorer quality of life (QoL). The platinum drug combinations with either gemcitabine, docetaxel or pemetrexed have reached a plateau offering a mean survival of approximately one year in advanced NSCLC [2]. Patients expressing immune checkpoint markers are amenable to treatment with monoclonal antibodies [3, 4]. The focus of NSCLC treatment shifted significantly with availability of inhibitors of targetable driver kinases such as mutated epidermal growth factor (EGFR) and anaplastic lymphoma kinase (ALK) rearrangements, among others [5].

The first-generation EGFR tyrosine kinase inhibitors (TKIs) gefitinib and erlotinib bind reversibly to the kinase domain of the receptor, but second-generation drugs such as the pan-ErbB inhibitor afatinib show irreversible inhibition of the kinase activity [6]. In NSCLC, pancreatic cancer and colorectal cancer, afatinib resulted in an inhibition of cellular growth and induction of apoptosis [7]. Although afatinib is most effective against mutated EGFR it is likewise active against the wildtype receptor. Unfortunately, the majority of NSCLC lacks actionable drivers and still have to be treated with cytotoxic combination chemotherapy. However, durable disease control is rare and the 5-year survival is below 5% [8]. Therefore, new agents with different mechanisms of antitumor activity may improve outcomes of NSCLC patients.

A range of antitumor compounds has been extracted from the sponge *Fascaplysinopsis spp*. with fascaplysin (12,13-dihydro-13-oxo-pyrido[1,2-a:3,4-b'] diindol-5-ium monochloride) as the most important agent [9]. Fascaplysin and derivatives exhibits a multitude of biological activities including antitumor effects based on antiproliferative and anti-angiogenic properties via targeting of cyclin-dependent kinase-4 (CDK4; IC_50_ 350 nM) and by intercalation into DNA [10, 11]. Furthermore, fascaplysin increases phosphorylation of Akt, and adenosine monophosphate-activated protein kinase (AMPK), which are essential due to their anti-apoptotic or pro-survival functions in cancer [12]. Fascaplysin inhibited ovarian cancer cell proliferation, invasion and migration and suppressed CDK4, cyclin D1, Bcl-2, and VEGF-A expression [13, 14].

Our previous studies revealed that fascaplysin exhibited high cytotoxicity against Small Cell Lung Cancer (SCLC) cell lines (mean IC_50_ 0.89 µM) and against SCLC Circulating Tumor Cell (CTCs) lines (mean IC_50_ 0.57 µM) [15, 16]. Selected NSCLC lines exhibited a mean IC_50_ of 1.15 µM for fascaplysin and the compound showed an additive cytotoxic effect with cisplatin. Available permanent cancer cell lines have been adapted for vigorous in vitro growth and may not be truly representative of the in vivo situation in patients. Acquisition of NSCLC cells for tests is possible by routine thoracentesis in patients with advanced NSCLC. Malignant pleural effusion (MPE) is observed in half of advanced NSCLC cases and is associated with a short survival [17]. MPE samples frequently contain numerous tumor cells, that allow for the determination of driver gene status and chemosensitivity [18–20]. In the present study, a panel of primary NSCLC lines from pleural effusions was employed to compare their chemosensitivity against fascaplysin with that for cisplatin. Furthermore, both drugs were combined with the afatinib to test a possible synergistic activity and with the Chk1/2 inhibitor AZD7762 to investigate DNA damage-mediated drug effects. The results demonstrate that afatinib acts synergistically with fascaplysin to sensitize the NSCLC cancer cells against this marine drug.

## Materials and methods

**Cell Culture and reagents** Unless otherwise noted, all chemicals were obtained from Sigma-Aldrich (St. Louis, MO, USA). Dulbecco’s phosphate buffered saline (PBS) was purchased from Gibco/Invitrogen (Carlsbad, CA, USA). Compounds were prepared as stock solutions of 2 mg/mL in either DMSO or 0.9% NaCl for cisplatin and aliquots stored at − 20 °C. Equivalent concentrations of DMSO were supplemented to medium controls. Established permanent cell lines were obtained from the American Type Culture Collection (Manassas, VA, USA) and primary lung cancer lines were established in our lab. Collection of pleural effusions of lung cancer patients, isolation of tumor cells and generation of cell lines was done according to the Ethics Approval 366/2003 by the Ethics Committee of the Medical University of Vienna, Vienna, Austria. In brief, pleural effusions were centrifuged and the tumor cells washed with tissue culture medium consisting of RPMI-1640 medium, supplemented with 10% FBS (Seromed, Berlin, Germany) and antibiotics (final concentrations: 50 U/mL of penicillin, 50 µg/mL of streptomycin, and 100 µg/mL neomycin). When required, erythrocytes were removed by Histopaque®-1077 (Sigma-Aldrich) gradient centrifugation. Primary NSCLC cell lines were established in tissue culture medium and cultures split by trypsination. All cell lines showed an EGFR del19 deletion, with exception of BH1059/RET mutation, BH419 BRCA1 mutation and three lines, namely BH482, BH583 and BH827, with ALK rearrangements.

**Phosphokinase Array** Relative protein phosphorylation levels of 38 selected proteins were obtained by analysis of 43 specific phosphorylation sites using the Proteome Profiler Human Phospho-Kinase Array Kit ARY003B/C (R&D Systems, Minneapolis, MN, USA) in duplicate tests carried out according to the manufacturer’s instructions. Briefly, cells were rinsed with PBS, 1 × 10^7^ cells/mL lysis buffer were solubilized under permanent shaking at 4 °C for 30 min, and aliquots of the lysates were stored frozen at − 80 °C. After blocking, membranes with spotted catcher antibodies were incubated with diluted cell lysates at 4 °C overnight. Thereafter, cocktails of biotinylated detection antibodies were added at room temperature for 2 h. Phosphorylated proteins were revealed using streptavidin-HRP/chemiluminescence substrate (SuperSignal West Pico, Thermo Fisher Scientific, Rockford, IL, USA) and detection with a Molecular Imager ChemiDoc MP imaging system (Bio-Rad, Hercules, CA, USA). Images were quantified using Image J (NIH, Bethesda, MD, USA) and Origin (OriginLab, Northampton, MA, USA) software. The different Western blot membranes were normalized using the 6 calibration spots included.

**Cytotoxicity Assay** Aliquots of 1 × 10^4^ cells in 200 µL medium were treated for four days with twofold dilutions of the test compounds in 96-well microtiter plates in quadruplicate (TTP, Trasadingen, Switzerland). The plates were incubated under tissue culture conditions and cell viability was measured using a modified MTT (3-(4,5-dimethylthiazol-2-yl)-2,5-diphenyltetrazolium bromide) assay (EZ4U, Biomedica, Vienna, Austria). Optical density was measured using a microplate reader at 450 nm and values obtained from control wells containing cells and media alone were set to 100% proliferation. For the assessment of the interaction of the test compounds, tests were performed comprising the individual drugs alone and in combination, followed by analysis using the Chou-Talalay method with help of the Compusyn software (ComboSyn Inc., Paramus, NJ, USA).

**Statistics** Statistical analysis was performed using Student’s t test for normally distributed samples (* p < 0.05 was regarded as statistically significant). Values are shown as mean ± SD.

## Results

### Cellular toxicity of fascaplysin, cisplatin and afatinib

**Fig. 1:**
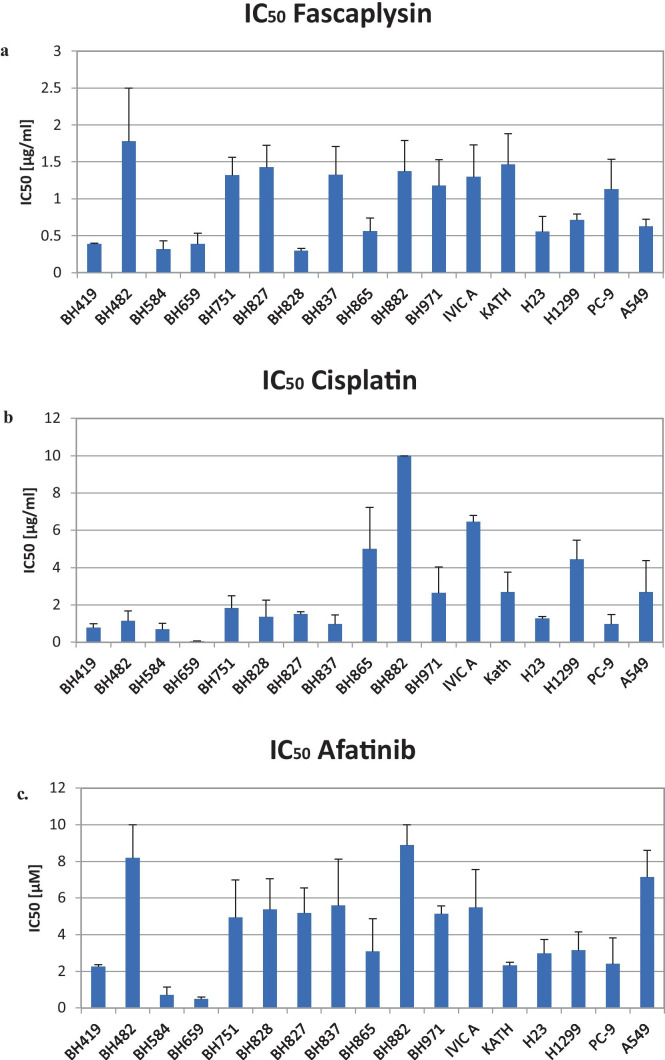
**a**-**c**. The figure show the IC_50_ values for fascaplysin (1**a**), cisplatin (1**b**) and afatinib (1**c**), for the panel of pleural primary NSCLC cell lines and H23, H1299, PC-9 and A549 permanent NSCLC lines, respectively. Data shown represent mean values ± SD

Cytotoxicity of fascaplysin, cisplatin and afatinib were determined in MTT assays employing primary NSCLC cell lines and the permanent NSCLC cell lines H23, H1299, PC9 and A549 (Fig. 1A-C). IC_50_ values for fascaplysin varied from 0.48 – 1.37 µg/ml, with 8/17 cell lines exhibiting high chemosensitivity (Fig. 1A). A group of cell lines with high sensitivity of 0.48 ± 0.14 µg/ml contrasts to a more resistant NSCLC cell population exhibiting a mean IC_50_ value of 1.37 ± 0.18 µg/ml (p = 0.001). The difference in fascaplysin sensitivity of the permanent cell line H23, H1299, PC-9 and A549 versus primary NSCLC lines is not statistically different.

The IC_50_ values for cisplatin show a distinct sensitivity pattern for the NSCLC cell lines tested (range: 1.42 – 6.48 µg/ml), with 13/17 cell lines exhibiting chemosensitivity below clinical achievable peak plasma concentrations (PPCs) of 3 µg/ml (Fig. 1B). A group with high sensitivity 1.42 ± 0.79 µg/ml contrasts to a more resistant NSCLC cell population with 6.48 ± 2.16 µg/ml (p = 0.001). Analysis of the fascaplysin and cisplatin IC_50_ values showed a lack of correlation (correlation coefficient r^2^ = 0.07) and, thus, completely different chemosensitivities of the primary NSCLC cells to these cytotoxic drugs.

In contrast, IC_50_ values for afatinib range from 2 µM to approximately 8 µM indicating relatively low sensitivity for these primary NSCLC cell lines with exception of BH584 and BH659 that have revealed a NSCLC-SCLC transformation (mean IC_50_: 4.81 ± 2.05 µM; Fig. 1C). Accordingly, several of these primary NSCLC lines have been obtained after progress under EGFR TKI therapy. The difference in afatinib sensitivity of the permanent cell line H23, H1299, PC-9 and A549 versus primary NSCLC lines is not statistically different. Due to high variability of the IC50 values observed for the permanent lines, differences to primary NSCLC lines were not significant for all drugs.

### Cellular toxicity of fascaplysin-afatinib combinations

**Fig. 2:**
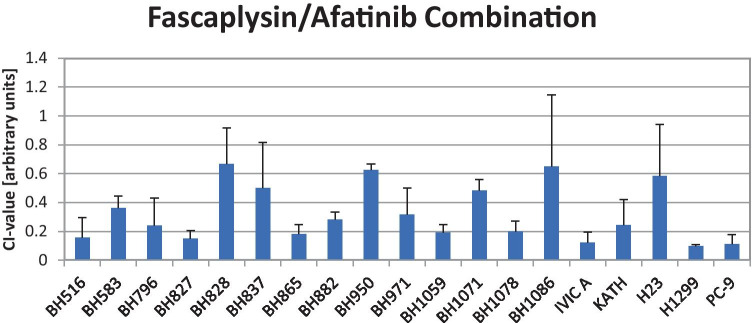
Overview of the CI values for the fascaplysin – afatinib combinations (mean values ± SD. Values below 1 indicate a synergistic interaction

The cytotoxic effects of fascaplysin-afatinib combinations were tested in proliferation assays using 10 twofold dilutions of the single drugs and a combination of the two drugs at full concentrations. The effects of the combinations were calculated according to the Chou-Talalay method. The combination indices (CIs) are shown in Fig. 2 and all tests revealed synergy of this combination with CIs ranging from 0.08 – 0.67. The mean CI value for the fascaplysin-afatinib combinations and all cell lines was 0.324 ± 0.19. For the three ALK-rearranged cell lines, the fascaplysin-alectinib and fascaplysin-crizotinib combinations were synergistic for BH482 and BH827 but not for the alectinib-resistant cell line BH583 (data not shown).

### Combinations of fascaplysin and cisplatin with AZD7762

**Fig. 3:**
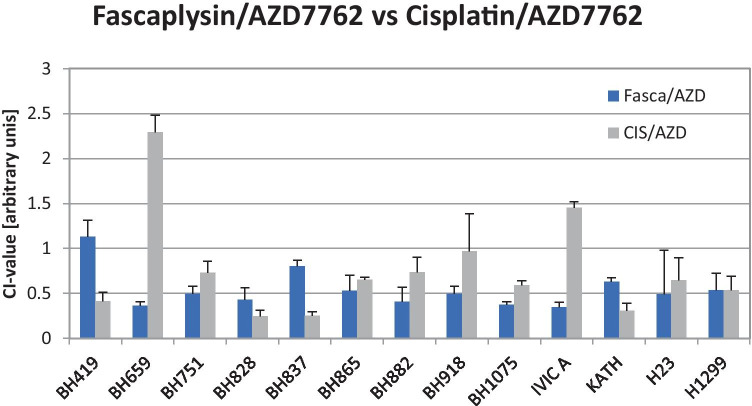
shows the CI values for the fascaplysin—AZD7762 combinations and the panel of NSCLC lines under investigation. Data represent mean values ± SD. Differences between fascaplysin/cisplatin AZD7762 were significant for all combinations, except for BH751, BH865, H23 and H1299

Combination experiments employing fascaplysin and AZD7762 showed synergistic effects with CI values ranging from 0.35 – 1.13 (mean value 0.54 ± 0.21), except of the BH419 BRCA1-mutated NSCLC cell line. The combinations of cisplatin with AZD7762 showed synergy in 3/13 cell lines (mean value 0.76 ± 0.53) with 10/13 cell lines being significantly different from fascaplysin-AZD7762 combinations (Fig. 3).

### Comparisonof IC_50_ values of fascaplysin-afatinib combinations versus fascaplysin single drug

**Fig. 4:**
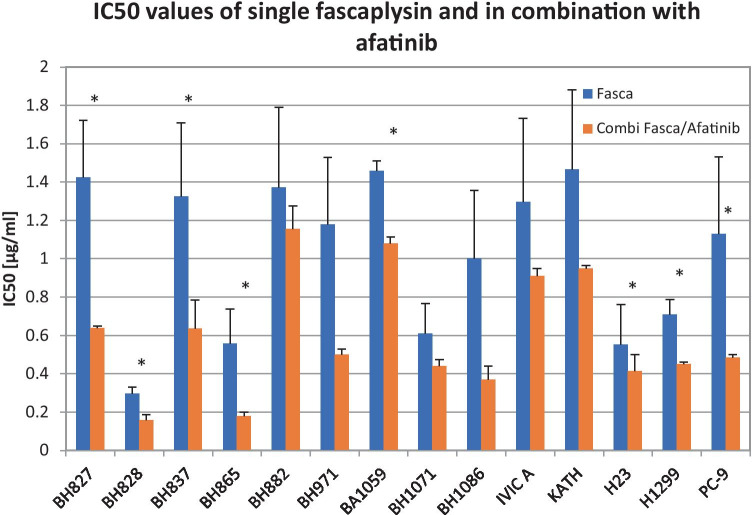
depicts the differences of the IC_50_ values of fascaplysin as single drug and in combination with afatinib. Data represent mean values ± SD and the statistically significant differences are marked with an asterisk

A comparison of the IC_50_ values of fascaplysin alone with IC_50_ values obtainted from fascaplysin-afatinib combinations showed significantly increased drug sensitivity of the NSCLC lines in 8/14 cases (Fig. 4).

### H1299 NSCLC cell line: effects of fascaplysin on protein phosphorylation

**Fig. 5:**
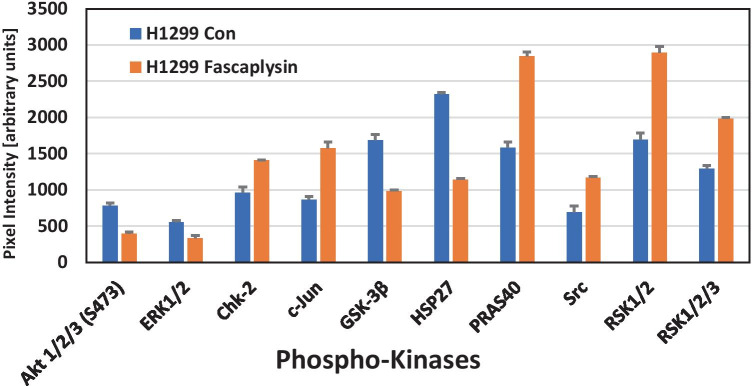
Out of the whole panel of phosphoproteins, the significantly altered proteins in fascaplysin-treated H1299 cells are presented in Fig. 5. Data presented are mean values ± SD of duplicate determinations. All differences betweem H1299 Con and H1299 Fascaplysin are statistically significant

Changes in the phosphorylation of signaling proteins of H1299 cells in response to fascaplysin were analyzed with help of a Western blot profiler array that detects 43 kinase phosphorylation sites and 2 related proteins. Significant changes in the phosphorylation pattern of selected proteins are shown in Fig. 5. Specific sites were hypophosphorylated for Akt1/2/3, ERK1/2, GSK-3β and HSP27, whereas Chk2, src, c-Jun, PRAS40 and RSK1/2/3 become hyperphosphorylated in response to drug exposure.

## Discussion

Therapy of NSCLC has changed dramatically with the advent of TKIs against driver kinases and the activation of antitumor immune responses by monoclonal checkpoint inhibitors (ICIs) [5]. However, the efficacy of such therapeutic modalities is restricted to approximately 30% of the patients and the majority of advanced NSCLC cases has still to be treated with cytotoxic chemotherapy. However, classical chemotherapy has reached a plateau at a low level in respect to overall survival (OS) [21]. The recent combinations of ICIs with chemotherapy revealed relatively low and unpredictable responses [22, 23]. Thus, novel compounds that hit targets different from that of the platinum-based combinations may improve responses and prolong survival. We have demonstrated previously that fascaplysin has high cytotoxic activity against SCLC, SCLC CTCs and a limited range of NSCLC lines [16]. Here, the chemosensitivity of a panel of primary pleural NSCLC lines against fascaplysin was compared to the cytotoxic effects of cisplatin. The IC_50_ values for fascaplysin ranged from 0.48 – 1.37 µg/ml for the whole NSCLC cell line panel and from 1.42 – 6.48 µg/ml for cisplatin, although most cell lines proved to be cisplatin-sensitive with IC_50_ values below and around 3 µg/ml. Thus, fascaplysin displays considerable cytotoxicity against the primary NSCLC lines that may be further boosted in combination with TKIs directed to EGFR.

The EGFR TKI afatinib is a second generation, irreversible ErbB family blocker, that exhibits inhibitory activity against EGFR, human EGFR 2 (HER2) and 4 (HER4), with IC_50_ values of 0.5, 14, and 1 nM, respectively [6, 24]. The IC_50_ afatinib values for the whole primary NSCLC cell line panel of 4.81 ± 2.05 µM is a typical result for cell lines not dependent on mutated EGFR, such as breast cancer cell lines T47D and BT20, whereas IC_50_ values for afatinib and cell lines addicted to mutated EGFR may be as low as 6–10 nM [25]. At extremes, NSCLC cell lines such as NCI-H460 and NCI-H226 exhibit afatinib IC_50_ values of approximately 50 µM. A pharmacokinetics analysis revealed that plasma concentrations of afatinib peaked at 3 – 4 h after administration and decreased with a half-life of 37 h at steady state [26]. Afatinib is administered at 40 mg PO/day resulting in approximately 0.2 µM peak plasma concentration after multiple dosing. Our results show that this TKI in combination with fascaplysin results in approximately twofold sensitization and a considerable decrease of the IC_50_ values. Although afatinib is the standard drug for the treatment of lung squamous cell carcinoma (SCC) with EGFR overexpression, attempts have been made to use this irreversible blocker for other EGFR expressing tumors. Advanced head and neck squamous cell carcinoma (HNSCC) hold a poor prognosis and tumor progression is associated with overexpression of EGFR [27]. Afatinib increased the cytotoxicity of cisplatin when combined in different schedules of exposure against these HNSCC cell lines. In detail, cisplatin treatment followed by afatinib exposure showed higher activity against two EGFR wildtype HNSCC cell lines compared to other approaches. Furthermore, EGFR was found hyperphosphorylated in cisplatin-resistant wildtype EGFR NSCLC cells, H358^R^ and A549^R^, and the cisplatin/gefitinib combination applied promoted apoptotic cell death [28]. Another study employing five human EGFR wild-type HNSCC cell lines showed significant synergy of afatinib with cisplatin [29]. In detail, in three out of the five cell lines 0.625 µM afatinib in combination with cisplatin exerted antiproliferative effects and the remaining two lines showed responses for a combination with ≥ 1.25 µM afatinib. Since the EGFR TKI gefitinib showed similar effects to afatinib in sensitizing wildtype EGFR NSCLC cells to cisplatin, the effects of afatinib seem not to be linked by off-target effects due to reactions with non-EGFR protein cysteine residues [30]. In general, the synergistic toxicity may be based on the link of EGFR signaling to the response to DNA damage by chemotherapeutic agents including cisplatin [31].

The induction of the DNA repair system involves sensing of the damage by ATM (ataxia-telangiectasia mutated) and ATR (ATM- and Rad3-Related) kinases and activation of Chk1/2 downstream kinases [32]. The overexpression of Chk1 is associated with poorer outcomes and may contribute to therapy resistance in NSCLC [33]. AZD7762 is a potent inhibitor of Chk1/2 that blocks specifically the ATP binding pocket (IC_50_ 5 nM) [34]. AZD7762 has activity on a range of other kinases SRC family members, colony stimulating factor receptor (CSF1R), RET and others. In combination with DNA-damaging agents such as gemcitabine, topotecan, doxorubicin, and cisplatin, AZD7762 inhibits cancer cell growth in vitro via Chk1 inhibition and abrogation of the G2 and S phase checkpoints [35]. The sensitzing effect of this inhibitor over the DNA-damaging agents alone ranged from 5- to 20-fold. Furthermore, AZD7762 could enhance cisplatin-mediated apoptosis by inhibiting damage repair in vitro and enhanced xenograft apoptosis induced by cisplatin in vivo [36]. Surprisingly, in our experiments the synergistic effect of AZD7762 on tumor cell death proved to be higher in fascaplysin-AZD7762 combinations versus cisplatin-AZD7762 combinations. Studies has shown that the intercalation of fascaplysin is regarded as the major binding mode for DNA [37]. Fascaplysin displaces ethidium bromide from DNA that is known to bind to the minor groove of doublestrand DNA and, therefore, intercalation is hold to be responsible for the unique cytotoxicity of native fascaplasin versus nonplanar derivatives and induction of the DNA repair system [38].

Investigation of fascaplysin-induced changes in protein phosphorylation in H1299 NSCLC cells was assessed using Western blot arrays, as previously demonstrated for the A549 cell line [16]. The PI3K/AKT/mTOR pathway, which plays essential roles in cell proliferation and survival is frequently deregulated in cancer, in particular due to loss of PTEN, as in the case of H1299 [39]. The fascaplysin-induced decreases in Akt (Ser473) phosphorylation are correlated with lower cell survival due to induction of apoptosis [40]. Decreased phosphorylation of the mitogen-activated protein kinase (MAPK) pathway terminal master kinases ERK1/2 results in diminished proliferation and was found here for the exposure to fascaplysin, [41]. Chk2 and Chk1 phosphorylation triggers DNA repair and hyperphosphorylation of c-Jun and Src which is linked to the cellular stress response [42]. Hypophosphorylation of multifunctional glycogen synthase kinase 3β (GSK3β) alters a key node of survival pathways mediated by Ser/Thr protein kinases related to Akt, protein kinase C (PKC), ERK1/2 and Wnt [43]. Furthermore, hypophosphorylation of the chaperone HSP27 is known to enhance the cytotoxicity of chemotherapeutics [44]. The p90 ribosomal S6 kinases (RSK1-4) comprise a family of serine/threonine kinases that lie at the terminus of the ERK pathway. RSKs promotes silencing of G2 DNA damage checkpoint in a Chk1-dependent manner, and activation of RSKs promotes resistance to DNA-damaging agents [45]. The cell stress response observed in H1299 seems to result in activation of the RSK kinases. The proline-rich Akt substrate of 40 kDa (PRAS40) is a substrate of Akt and is phosphorylated by growth factors or other stimuli. PRAS40 is an important substrate of the Akt3 kinase, which regulates the apoptotic sensitivity of cancer cells and becomes activated in H1299 to counteract the cytotoxic effects of fascaplysin [46]. The fascaplysin-induced alterations in protein phosphorylation indicate efficient execution of cytotoxic effects and a failing intracellular stress response.

In summary, fascaplysin promotes cell death of NSCLC cell line in a manner different from the standard platinum drugs. This marine drug induces a DNA repair response, syngergizes with the Chk1/2 inhibitor AZD7762 and with the EGFR TKI afatinib.

## Data Availability

All data and materials are available under reasonable request.
